# Deferoxamine Counteracts Cisplatin Resistance in A549 Lung Adenocarcinoma Cells by Increasing Vulnerability to Glutamine Deprivation-Induced Cell Death

**DOI:** 10.3389/fonc.2021.794735

**Published:** 2022-01-20

**Authors:** Wen-Jun Liu, Peng-yu Pan, Ye Sun, Jian-bo Wang, Huan Zhou, Xin Xie, Zhi-yuan Duan, Han-yu Dong, Wen-na Chen, Li-de Zhang, Chun Wang

**Affiliations:** ^1^ Teaching and Experimental Center, Liaoning University of Traditional Chinese Medicine, Shenyang, China; ^2^ Department of Cell Biology, College of Integrated Chinese and Western Medical, Liaoning University of Traditional Chinese Medicine, Shenyang, China; ^3^ Key Laboratory of Environmental Pollution and Microecology of Liaoning Province, Shenyang Medical College, Shenyang, China; ^4^ Key Laboratory of Ministry of Education for Traditional Chinese Medicine (TCM) Viscera-State Theory and Applications, Liaoning University of Traditional Chinese Medicine, Shenyang, China

**Keywords:** NSCLC, cisplatin resistance, glutamine deprivation, metabolic reprogramming, deferoxamine, cell death

## Abstract

Glutamine, like glucose, is a major nutrient consumed by cancer cells, yet these cells undergo glutamine starvation in the cores of tumors, forcing them to evolve adaptive metabolic responses. Pharmacologically targeting glutamine metabolism or withdrawal has been exploited for therapeutic purposes, but does not always induce cancer cell death. The mechanism by which cancer cells adapt to resist glutamine starvation in cisplatin-resistant non-small-cell lung cancer (NSCLC) also remains uncertain. Here, we report the potential metabolic vulnerabilities of A549/DDP (drug-resistant human lung adenocarcinoma cell lines) cells, which were more easily killed by the iron chelator deferoxamine (DFO) during glutamine deprivation than their parental cisplatin-sensitive A549 cells. We demonstrate that phenotype resistance to cisplatin is accompanied by adaptive responses during glutamine deprivation partly *via* higher levels of autophagic activity and apoptosis resistance characteristics. Moreover, this adaptation could be explained by sustained glucose instead of glutamine-dominant complex II-dependent oxidative phosphorylation (OXPHOS). Further investigation revealed that cisplatin-resistant cells sustain OXPHOS partly *via* iron metabolism reprogramming during glutamine deprivation. This reprogramming might be responsible for mitochondrial iron-sulfur [Fe-S] cluster biogenesis, which has become an “Achilles’ heel,” rendering cancer cells vulnerable to DFO-induced autophagic cell death and apoptosis through c-Jun N-terminal kinase (JNK) signaling. Finally, *in vivo* studies using xenograft mouse models also confirmed the growth-slowing effect of DFO. In summary, we have elucidated the adaptive responses of cisplatin-resistant NSCLC cells, which balanced stability and plasticity to overcome metabolic reprogramming and permitted them to survive under stress induced by chemotherapy or glutamine starvation. In addition, for the first time, we show that suppressing the growth of cisplatin-resistant NSCLC cells *via* iron chelator-induced autophagic cell death and apoptosis was possible with DFO treatment. These findings provide a solid basis for targeting mitochondria iron metabolism in cisplatin-resistant NSCLC for therapeutic purposes, and it is plausible to consider that DFO facilitates in the improvement of treatment responses in cisplatin-resistant NSCLC patients.

## Introduction

Lung cancer is the leading cause of cancer-related deaths worldwide. Non-small-cell lung cancer (NSCLC) represents 85% of lung cancer cases. Although advances in treatment of NSCLC have been facilitated by better understanding of pathogenic genomic alterations of NSCLC, cytotoxic chemotherapy remains an important component of systemic therapy for the majority of patients. Cisplatin is one of the most widely used chemotherapeutic agents; however, cisplatin resistance has become a major obstacle in clinical oncology (1–3). Deoxyribonucleic acid (DNA) has been thought to be the primary target of cisplatin, but recent studies have shown that only a very tiny percentage of cisplatin (1%) interacts with nuclear DNA (4–7). Instead, most cisplatin interacts with mitochondria, which in turn has revealed the fundamental role of mitochondria in chemotherapy resistance (8, 9).

Mitochondria integrate catabolism, anabolism, and signaling (9). While originally relegated to the role of the “energy powerhouse” of the cell, mitochondria are now well established as the hub of numerous signaling pathways that have been implicated in most cellular processes (10). In fact, in addition to their role in the Krebs cycle, oxidative phosphorylation (OXPHOS), and fatty-acid oxidation (FAO), mitochondria are also involved in the intrinsic apoptotic signaling pathway, thereby governing cell death (11). These functions enable mitochondria to sense cellular stress, which allows them to confer a high level of plasticity to cells, thereby permitting rapid adaptation to challenging microenvironmental conditions (2, 12).

Cell reprogramming faces the challenges of balancing stability with plasticity and of overcoming critical barriers such as cell cycle checkpoints, mesenchymal-epithelial transition (MET), and metabolic reprogramming (12, 13). Alterations in cellular metabolism have emerged as a hallmark of cancer (14). Numerous studies have shown that cisplatin-resistant cells acquire drug resistance and undergo a major rewiring of their metabolism, as revealed by changes in enzymes that are involved in glucose and glutamine metabolism (3, 15, 16). Glutamine contributes to multiple biosynthetic pathways, supports the Krebs cycle and OXPHOS, and governs redox homeostasis (17, 18). Therefore, cancer cells are highly dependent on glutamine, and their deprivation thereof leads to severe cell death. Consequently, cancer cells mount complicated adaptive responses to ensure their survival, and these responses are not fully understood (1, 14, 15).

Iron is a metal micronutrient that is required for basic energy metabolism, mitochondrial function, and DNA synthesis (19, 20). In respiring cells, iron plays crucial roles in the synthesis of [Fe-S] clusters and drives electron transfer pathways in mitochondrial respiratory complexes (21). Colorectal cancer (CRC) requires massive iron stores relative to adjacent normal cells (22). Iron uptake and dependence are enhanced in cancer stem-like cells (CSCs) (23). However, how cisplatin-resistant NSCLC modulates the local iron supply remains unclear. In this study, we report on the potential metabolic vulnerabilities of cisplatin-resistant A549 lung adenocarcinoma (LAD) cells, which are resistant to glutamine deprivation while being more easily killed by the iron chelator deferoxamine (DFO) during glutamine deprivation than their parent cisplatin-sensitive A549 cells. We also provide evidence that targeting this compensatory response could be an important therapeutic strategy against cancer cells.

## Materials and Methods

### Reagents and Antibodies

The main reagents used in this study included L-glutamine (#C0212; Beyotime Institute of Biotechnology, Haimen, China) and cisplatin (PHR1624; Sigma Germany, Munich, Germany). Matrigel™ Basement Membrane Matrix (#356234) was obtained from Corning and pentobarbital sodium from Merck. A siRNA kit targeted to SDHB (stB0007812A) was purchased from RiboBio Technology Co., Ltd. (Guangzhou, China). A Golden Trans DR Reagent (#PE-401-3001) was obtained from Golden Trans Technology Co., Ltd. (Changchun, China). A cell counting kit-8 (CCK-8; #C0037), an adenosine triphosphate (ATP) assay kit (#S0026), a mitochondrial membrane potential (MMP) assay kit with tetraethylbenzimidazolylcarbocyanine iodide (JC-1; #C2006), and a reactive oxygen species (ROS) assay kit (#S0033S) were also bought from Beyotime. We purchased a reduced glutathione (GSH) assay kit (#A006) from Nanjing Jiancheng Bioengineering Institute Co., Ltd. (Nanjing, China) and an iron colorimetric assay kit (#E-BC-K139-S) from Elabscience (Houston, TX, USA). DFO (#Y0001934) was obtained from the European Directorate for the Quality of Medicines & HealthCare (EDQM; Strasbourg, France). From MedChemExpress (Monmouth Junction, NJ, USA), we purchased Z-VAD-fluoromethylketone (Z-VAD-fmk; #HY-166588), bafilomycin A1 (BA1; #HY-100558), and DB07268 (#HY-15737). C11-BODIPY^581/591^ (#D3861) was obtained from Invitrogen Corp. (Carlsbad, CA, USA). Agilent Technologies, Inc. (Santa Clara, CA, USA) was our source for Seahorse XF Dulbecco’s modified Eagle’s medium (DMEM; #103575), a Seahorse XF Mito Fuel Flex Test Kit (#103260), and a Seahorse XF Cell Mito Stress Test Kit (#103010). From Cell Signaling Technology (CST; Danvers, MA, USA), we purchased primary antibodies, such as those against ferritin-heavy polypeptide (FTH; #4393), divalent metal transporter 1 (DMT1; #15083), hexokinase 1 (HK1; #2804), HK2 (#2106), and pyruvate kinase muscle isozyme M2 (PKM2; #3198). Antibodies against SDHB (#10620-1-AP), caspase-3 (#19677-1-AP), B-cell lymphoma 2 (*Bcl-2*; #12789-1-AP), *Bcl-2*-like protein 4 (*Bax*; #50599-2-Ig), p62/sequestosome-1 (SQSTM1; #18420-1-AP), ferroportin (Fpn; #26601-1-AP) and β-actin (#66009-1-Ig) were obtained from Proteintech (Chicago, IL, USA). From Abcam (Cambridge, UK), we procured cleaved caspase-3 (#ab32042), light chain 3B (LC3B; #48394), and total OXPHOS complex (#110413). Transferrin receptor protein 1 (TfR-1; #13-6800) was obtained from Invitrogen. From Bioss Antibodies (Woburn, MA, USA), we obtained phospho-P38 mitogen-activated protein kinase (MAPK; Thr180; #5476), phosphor-extracellular signal-regulated kinase 1/2 (ERK1/2; Thr202 + Tyr204; #3016), and phospho-JNK1/2/3 (T183+T183+T221; #4163). We detected electrochemiluminescent (ECL) bands in western blotting (WB) using an ECL detection reagent (#1805001; Tanon Science & Technology Co., Ltd., Shanghai, China).

### Cell Culture, Glutamine Deprivation, DFO Treatment, and Inhibitor Treatment

We purchased both the A549 and A549/DDP cell lines from Beijing Dingguo Changsheng Biotechnology Company, Ltd. (Beijing, China) and used these within three months of thawing. Cells were tested for *Mycoplasma* using a One-step Quickcolor *Mycoplasma* Detection Kit (#MD001; Shanghai YISE, Shanghai, China). We cultured both types of cells in glutamine-free DMEM/Nutrient Mixture F-12 [DMEM/F12; #PM150313; ProCell Therapies (Clarion Medical Technologies; Cambridge, ON, Canada)] supplemented with L-glutamine (2 mM), 10% fetal bovine serum (FBS; #FS301-02; TransGen Biotech, Inc., Beijing, China), 100 units/mL penicillin, and 100 mg/mL streptomycin solution [#SV30010; HyClone (GE Healthcare, Chicago, IL, USA)] at 37°C in a 5% CO_2_ incubator. Drug-resistant A549/DDP cells were maintained in complete medium containing 2 mg/mL cisplatin without penicillin-streptomycin antibiotics; the drug was withdrawn two weeks before the experiment.

For the glutamine deprivation assay, we cultured cells in complete medium without 2 mM glutamine for 48 h. For DFO treatment, cells were incubated in culture medium containing DFO (100 μM) for 48 h. Apoptosis/autophagy and JNK1 inhibitors were applied as follows: Z-VAD-fmk, 5 μM; BA1, 10 nM; DB07268, 10 nM.

### Cell Viability Assay

We assessed cell viability using the CCK-8 assay. Cells were seeded in 96-well microplates at a density of 5 × 10^3^ cells/well. After overnight incubation, we treated the cells with conditioned medium for 24 h or 48 h. Cell growth was assessed under a microscope before the CCK-8 assay. Subsequently, 10 μL of CCK8 solution were added to each well. After incubation for 1 h at 37°C, we used a Tecan Infinite 200 PRO microplate reader (Tecan Group, Ltd., Männedorf, Switzerland) to measure the optical density (OD) at a wavelength of 450 nm.

### Annexin V-FITC/PI Apoptosis Assay

To check the cell apoptosis rate, we performed an Annexin V-fluorescein isothiocyanate (FITC)/propidium iodide (PI) assay (#211-01; Vazyme Biotech Co., Ltd., Nanjing, China) using a BD Accuri C6a flow cytometer (FCM; BD Biosciences, Franklin Lakes, NJ, USA) as per manufacturers’ protocols. The results were represented as dot plots that were created using FlowJo software version 10.7 (BD Biosciences). Using unstained control cells, we gated the FCM dot plot data.

### Transmission Electron Microscopy

Cells were fixed with 2.5% glutaraldehyde in 0.1 M phosphate buffer for 30 min at room temperature (RT) and post-fixed in 1% osmium tetroxide in the same buffer for 1 h at RT. After dehydrating them through a graded series of ethanol, we embedded the samples in EPON resin (Hexion, Inc., Columbus, OH, USA). Ultrathin sections were stained with 2% uranyl acetate and lead citrate. We viewed the sections under a Hitachi JEM-1200EX transmission electron microscope (TEM; Hitachi, Tokyo, Japan).

### Immunofluorescence Staining

Cells were seeded onto coverslips in a 24-well plate. After overnight incubation, we treated the cells with conditioned medium for 48 h. After removal of the medium, cells were washed in phosphate-buffered saline (PBS) (#SH30256.01; HyClone) and fixed with cold 4% paraformaldehyde for 15 min. Following permeabilization with methyl alcohol for 10 min, we incubated the cells in QuickBlock Blocking Buffer for Immunol Staining (#P0260; Beyotime) serum for 15 min and then in rabbit anti-LC3B antibody (1:250; Abcam) overnight. Subsequently, cells were treated with AlexaFluor594 goat anti-rabbit immunoglobulin G (IgG; 1:400; Proteintech) for 1 h at RT in the dark and counterstained with antifade mounting medium containing 4’,6-diamidino-2-phenylindole (DAPI; #P0131; Beyotime) for 5 min. We captured photographs under an inverted fluorescence microscope (ZOE Fluorescent Cell Imager; Bio-Rad Laboratories, Hercules, CA, USA).

### siRNA Transfection

The A549/DDP cells were plated at a density of 5 × 10^3^ cells per well in a 96-well plate and cultured overnight. Then, the cells were transfected 24 h prior to glutamine deprivation with siRNA-targeting SDHB (stB0007812C, sequence 5’→3’: CCCGAAGGTTGACACCAA) or non-targeting control (RiboBio Technology) using Golden Trans DR Reagent. After treatment for 48 h, cell viability was assessed using a CCK-8 assay, and the knockdown of SDHB was confirmed by immunoblot analysis according to the manufacturer’s instructions.

### Oxygen Consumption Measurement

We calculated cell glutamine or glucose dependency using the Seahorse XF Mito Fuel Flex Test Kit on a Seahorse XFe96 Analyzer (Agilent) as per the manufacturer’s instructions. Briefly, A549 and A549/DDP cells were seeded in XFe96-well microplates at a density of 8 × 10^3^ cells/well in 100 μL of DMEM/F12 medium and placed in a 37°C incubator with 5% CO_2_ overnight. We replaced the medium with 180 μL assay medium containing glutamine (2 mM), sodium pyruvate (1 mM), and glucose (10 mM), pre-warmed to 37°C. Cells were incubated at 37°C without CO_2_ for 60 min to allow them to pre-equilibrate with the assay medium before the first measurement. After the equilibration period, we subjected the cells to four baseline measurements, followed by injection of *bis*-2-(5-phenylacetamido-1,3,4-thiadiazol-2-yl)ethyl sulfide (BPTES; 3 μM) and Eto (4 μM)/UK5099 (2 μM) for the glutamine dependency assay and UK5099 (2 μM) and Eto (4 μM)/BPTES (3 μM) for the glucose dependency assay. The dependency rate (the measure of cells’ reliance on the glutamine oxidation pathway to maintain baseline respiration) was calculated using the following equations (OCR = oxygen consumption rate): Glutamine dependency (%) = [(BPTES OCR - Eto/UK5099 OCR)/(BPTES OCR - All inhibitors OCR)] × 100% Glucose dependency (%) = [(UK5099 OCR - Eto/BPTES OCR)/(UK5099 OCR - All inhibitors OCR)] × 100%.

We measured the OCR on the Seahorse XFe96 Analyzer as per manufacturer’s instructions. Briefly, A549 and A549/DDP cells were cultured in DMEM/F12 with or without 2 mM glutamine for 30 h, seeded into XFe96-well microplates at a density of 8 × 10^3^ cells/well in 100 μL, and placed in a 37°C incubator with 5% CO_2_ overnight. We initiated assays at 48 h by replacing the medium with 180 μL of assay medium (2 mM glutamine, 1 mM sodium pyruvate, 10 mM glucose; pH 7.4) pre-warmed to 37°C. Cells were incubated at 37°C without CO_2_ for 1 h to allow these to pre-equilibrate with the assay medium before the first measurement. After the equilibration period, cells were subjected to three baseline measurements, followed by injection of the following reagents: 1.5 μM oligomycin, an ATP synthesis inhibitor, to assess O_2_ consumption devoted to ATP synthesis; 1 μM carbonyl cyanide-4-(trifluoromethoxy) phenylhydrazone (FCCP), an uncoupling agent, to measure uncoupled respiration; and 0.5 μM rotenone, a complex I inhibitor, to assess complex I -linked respiration.

### Determination of ATP

We measured ATP levels using an ATP assay kit (Beyotime). Briefly, cells were scraped off and homogenized on ice in ATP lysis buffer. After centrifugation at 12,000 g and 4°C for 5 min, we reserved the supernatant. We added 100 μL ATP detection working solution to each well of a black microwell plate at RT for 5 min to exhaust the background. Then, 20 μL of sample or standard was added to each well and immediately mixed. Finally, we measured luminescence using a SpectraMax i3X luminometer (Molecular Devices, Sunnyvale, CA, USA).

### Iron Colorimetric Assay

We measured cellular iron concentration using the Elabscience iron colorimetric assay kit. Briefly, cells were scraped off and homogenized on ice in PBS. After centrifugation, we reserved the supernatant and quantified it using a bicinchoninic acid (BCA) protein assay kit (#P0012; Beyotime). Next, we mixed 600 μL chromogenic agent with 200 μL sample and determined the OD at a wavelength of 520 nm using a Tecan Infinite 200 PRO microplate reader.

### Lipid Peroxidation Assay

Cells were seeded at 4 × 10^4^ cells/mL in a six-well plate and cultured with or without glutamine (2 mM) for 48 h. Then, we substituted the culture medium with 500 μL C11-BODIPY^581/591^ dye, which we dissolved in serum/phenol red-free base medium. After 30 min of incubation at 37°C, the cells were imaged under a ZOE Fluorescent Cell Imager.

### ROS Assay

ROS was determined by staining cells with DCFH-DA (#S0033S; Beyotime). Briefly, cells were washed with PBS solution twice and then incubated with DCFH-DA dyeing buffer for 20 min at 37°C. The ratios of DCF fluorescence were quantified on the BD Accuri C6.

### Mitochondrial-Membrane Potential Assay

MMP was determined by staining cells with JC-1 (#C2006; Beyotime). Briefly, cells were washed with PBS solution twice and then incubated with JC-1 dyeing buffer for 20 min at 37°C. We viewed and captured representative images under the ZOE Fluorescent Cell Imager. The relative ratios of red and green fluorescence were also quantified on the BD Accuri C6.

### Western Blot Analysis

Cells were washed twice with precooled PBS solution and lysed with radioimmunoprecipitation assay (RIPA) lysate (#P0013B, Beyotime) containing a protease inhibitor mixture (200 mM 4-benzenesulfonyl fluoride hydrochloride [AEBSF], 30 µM aprotinin, 13 mM bestatin, 1.4 mM E64, and 1 mM leupeptin). We measured protein concentrations using the BCA protein assay kit. Lysate samples with equivalent protein concentrations were separated *via* sodium dodecyl sulfate polyacrylamide gel electrophoresis (SDS-PAGE) and electrotransferred to PVDF membranes (0.25 µm; Bio-Rad Laboratories, Hercules, CA, USA). We blocked the membranes with 5% BSA in Tris-buffered saline + Polysorbate 20 (TBST) for 1 h at RT and then incubated these with the appropriate primary antibodies overnight at 4°C. Following further incubation with the appropriate secondary antibodies for 1 h at RT, we detected immune complexes using ECL reagent. Protein bands were visualized using a Tanon 5200 imaging system (Tanon Science & Technology Co., Ltd., Shanghai, China) and analyzed using AlphaView software version 3.4.0.0 (ProteinSimple, San Jose, CA, USA). We quantified relative protein levels by normalization to the internal reference protein β-actin.

### Animal Studies

BALB/c nude mice (6 weeks old, 8 females and 8 males) were obtained from Beijing HFK Bioscience Co., Ltd. (Beijing, China). After three days of acclimatization in a 7.6-L independent ventilation cage (IVC) (Shinva Medical Instrument Co., Ltd.) (Zibo, China), 16 BALB/c-nu mice were injected subcutaneously with 2 × 10^6^ A549/DDP cells and then randomized into Control and DFO treatment groups and treated intraperitoneally with PBS or DFO (8 mg/kg) twice per week in the vertical laminar flow clean workbench (Shinva Medical Instrument Co., Ltd. (BSE-CC-A 1000). Mice were treated for three weeks before the animals were humanely sacrificed (1% pentobarbital sodium dissolved in PBS for anesthetization) and the tumors were excised and weighed. This study’s animal ethics were approved by the Animal Core & Welfare Committee of Liaoning University of Traditional Chinese Medicine (No. 21000042021107).

### Statistical Analysis

Data were expressed as mean ± standard deviation (SD). We analyzed differences between two groups using two-tailed student’s *t* tests. ANOVA was used to compare multiple groups of data to determine differences among groups. *P* < 0.05 was chosen to indicate a statistically significant difference. We used SPSS software version 19.0 (IBM Corp., Armonk, NY, USA) for statistical analysis and GraphPad Prism software version 7.0 (GraphPad Software, Inc., San Diego, CA, USA) for charting.

## Results

### Cisplatin-Resistant Lung Adenocarcinoma Cells Attain Phenotype Resistance to Cisplatin Followed by Enhanced Adaptation During Glutamine Deprivation

**Figure 1:**
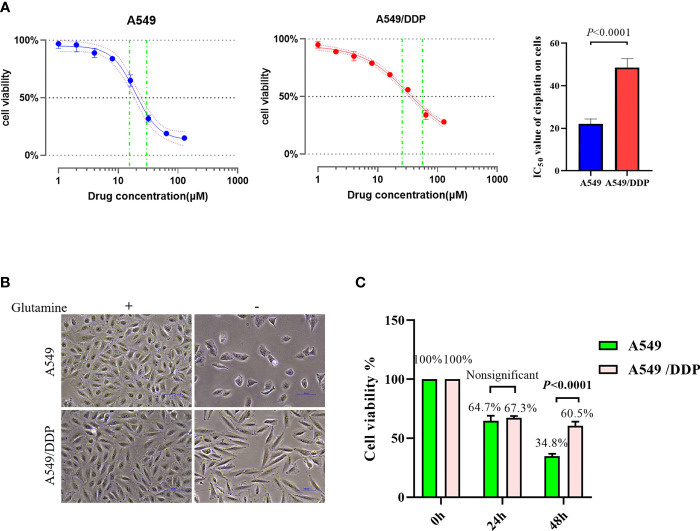
A549/DDP cells attain phenotype resistance to cisplatin as well as acquire an adaptation mechanism during glutamine deprivation. **(A)** Inhibition rate of A549 and A549/DDP cells after 24 h of cisplatin treatment (0, 2, 4, 8, 16, 32, 64, and 128 μM), as well as IC_50_ values of cisplatin in A549 and A549/DDP cells, are respectively represented by a line chart and a histogram. **(B)** Morphological observation of A549 and A549/DDP cells cultured with (+) or without (−) glutamine (2 mM; 48 h). **(C)** Cell viability assay 0, 24, and 48 h after glutamine deprivation in both cell lines. Experiments were repeated at least three times, and results were expressed as mean ± SD.

To confirm cisplatin resistance, we treated the parental cisplatin-sensitive A549 cells and cisplatin-resistant A549/DDP cells with different concentrations of cisplatin, and their IC_50_ values were 21.33 and 49.51 μM, respectively (**Figure 1A**). Next, to investigate the effect of glutamine deprivation on cell viability, we cultured A549 and A549/DDP cells with or without glutamine for 48 h and then assessed cell morphology and survival. The A549 cells demonstrated shattered and irregular shapes, whereas A549/DDP cells were elongated (**Figure 1B**). Of note, no differences in cell viability were observed after glutamine deprivation for 24 h between the two cell lines; nevertheless, the number of A549/DDP cells was more than the number of A549 cells did at 48 h (**Figure 1C**). Taken together, these results indicate that cisplatin-resistant cells not only achieved phenotype resistance to cisplatin, but also acquired an adaptation mechanism during glutamine deprivation.

### Cisplatin-Resistant Lung Adenocarcinoma Cells Display Higher Levels of Autophagic Activity and More Apoptosis Resistance Characteristics During Glutamine Deprivation

**Figure 2:**
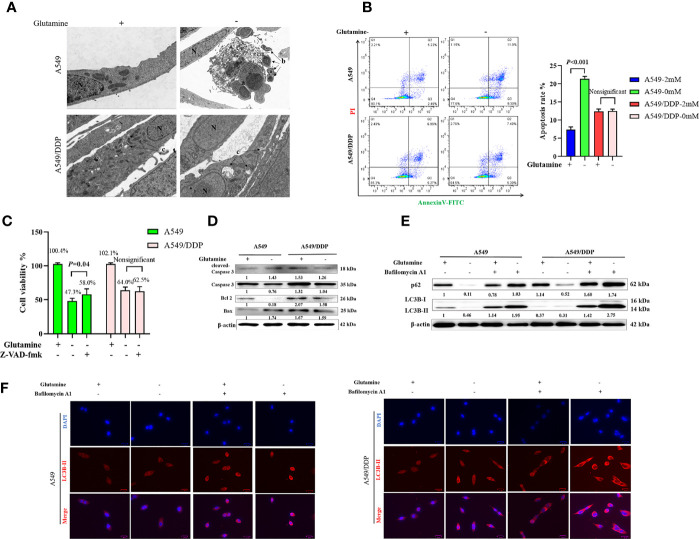
A549/DDP cells show higher levels of autophagic activity and greater apoptosis resistance characteristics during glutamine deprivation. **(A)** TEM images of A549 and A549/DDP cells cultured with (+) or without (−) glutamine (2 mM) for 48 h. a, an apoptotic cell with two nuclear fragments; b, apoptotic bodies; c, autophagosomes or initial autophagic vacuoles; N, nucleus. Bar, 2 μm. **(B)** Annexin V-FITC/PI apoptosis detection assay for A549 and A549/DDP cells cultured with (+) or without (−) glutamine (2 mM) for 48 h. **(C)** Effects of Z-VAD-fmk (5 μM; 48 h) on A549 and A549/DDP cells during 48 h of glutamine deprivation. **(D)** Blots show expression of key proteins involved in apoptosis in A549 and A549/DDP cells cultured with (+) or without (−) glutamine (2 mM) for 48 h. β-actin served as a loading control. **(E)** Blots show expression of key proteins involved in autophagy in A549 and A549/DDP cells after BA1 treatment (10 nM; 48 h) cultured with (+) or without (−) glutamine (2 mM) for 48 h. β-actin served as a loading control. **(F)** Immunostaining of LC3B in A549 and A549/DDP cells after BA1 treatment (10 nM; 48 h) cultured with (+) or without (−) glutamine (2 mM) for 48 h. Red = LC3B; blue = DAPI. All experiments were repeated at least three times, and results were expressed as mean ± SD.

We performed TEM at an ultrastructural level. Autophagosomes and apoptotic formation differed notably between the two cell lines. We observed the formation of more autophagosomes or initial autophagic vacuoles accompanied by fewer apoptotic bodies in A549/DDP cells than in A549 cells after glutamine deprivation for 48 h (**Figure 2A**). To further characterize glutamine withdrawal-induced cell death between the two cell lines, we performed the Annexin V-FITC/PI apoptosis detection assay on the FCM, and the results showed that glutamine deprivation for 48 h notably elevated the apoptosis rate in A549 cells, but made no difference in A549/DDP cells (**Figure 2B**). These results were further confirmed by the CCK-8 assay. We found that the apoptosis inhibitor Z-VAD-fmk (5 μM; 48 h) could rescue glutamine deprivation–induced cell death in A549 cells, but had no effect on A549/DDP cells (**Figure 2C**). Furthermore, we observed that glutamine deprivation inhibited anti-apoptosis protein *Bcl-2* expression while promoting pro-apoptosis protein *Bax* expression and then Caspase-3 activation in A549 cells. A549/DDP cells exhibited the opposite apoptosis profile: notably decreased *Bax* and cleaved Caspase-3 protein expression, despite accompaniment with a slight decrease in *Bcl-2* expression (**Figure 2D**). Collectively, these data implied that cisplatin-resistant LAD cells showed apoptosis resistance characteristics during glutamine deprivation.

LC3B is an acknowledged marker of autophagosome formation. SQSTM1/p62 is a multifunctional adaptor protein implicated in selective autophagy (24). After blocking the late stage of autophagy using BA1 (10 nM; 48 h), we showed that glutamine deprivation for 48 h increased LC3B-II and p62 protein expression in both cell lines. More importantly, A549/DDP cells showed more LC3B-II and p62 protein expression than A549 cells in glutamine-containing medium, which indicated an elevated basal autophagic activity that might have been responsible for the acquired cisplatin resistance. Similarly, glutamine deprivation for 48 h potently initiated autophagy in A549/DDP cells while moderately affecting A549 cells, as demonstrated by the notable elevation of LC3B-II and p62 protein expression in A549/DDP cells compared with A549 cells after BA1 treatment (10 nM; 48 h; **Figure 2E**). LC3B immunostaining results were also in accordance with our WB results: we found more LC3B puncta in A549/DDP than in A549 cells whether in glutamine-containing or -deprived medium (**Figure 2F**). Therefore, our results showed that A549/DDP cells displayed higher levels of autophagic activity during glutamine deprivation.

Collectively, these data implied that cisplatin-resistant LAD cells showed higher levels of autophagic activity and more apoptosis resistance characteristics during glutamine deprivation.

### Complex II-Dependent OXPHOS Triggers Adaptive Responses During Glutamine Deprivation and the SDHB Subunit Can be Utilized as Indicator of Drug Resistance in Lung Cancer

**Figure 3:**
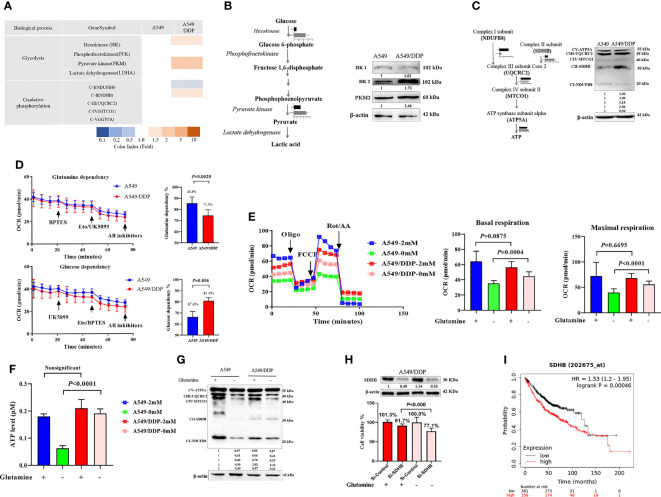
Complex II-dependent OXPHOS drives the adaptive response during glutamine deprivation, and the SDHB subunit can be exploited as a vulnerability index of drug resistance in lung cancer. **(A)** Gene transcripts of glycolysis and respiration complexes generated by RNA-Seq analysis in A549 and A549/DDP cells cultured with 2 mM glutamine. **(B, C)** Blots of glycolysis and respiration complexes in A549 and A549/DDP cells cultured in basal medium containing glutamine (2 mM). β-actin served as loading control. **(D)** The glutamine dependency rate (measure of cell reliance on the glutamine oxidation pathway to maintain baseline respiration) was calculated using the following equation: Dependency% = [(BPTES OCR − Eto/UK5099 OCR)/(BPTES OCR − all inhibitors OCR)] × 100%. **(E)** Oxygen consumption was represented by basal and maximal respiration as measured by Seahorse XF assay in A549 and A549/DDP cells cultured with (+) or without (−) glutamine for 48 h. **(F)** ATP levels of A549 and A549/DDP cells cultured with (+) or without (−) glutamine for 48 h. **(G)** Blots of respiration complexes in A549 and A549/DDP cells cultured with (+) or without (−) glutamine for 48 h. β-actin served as loading control. **(H)** Blots of gene-silencing SDHB (succinate dehydrogenase [ubiquinone] iron-sulfur subunit, mitochondrial) in A549/DDP cells and cultured with (+) or without (−) glutamine for 48 h using si-RNA. β-actin served as a loading control. Cell viability was monitored using a CCK-8 assay. Data were expressed as the mean ± SD, and the si-Control was set as 100% in glutamine-containing or glutamine-deprived medium of a representative experiment. **(I)** Kaplan–Meier analysis of overall survival of lung adenocarcinoma patients (n = 718) with respect to SDHB expression, demonstrating that higher expression is correlated with worse overall survival. All experiments were repeated at least three times, and results were expressed as mean ± SD.

Our previous reports showed that cisplatin-resistant A549/DDP cells showed higher levels of aerobic glycolysis than cisplatin-sensitive A549 cells, characterized by elevated key enzyme protein expression and enzymatic activity that modulate glycolysis (3, 16). In the present study, we also found a huge difference in the messenger RNA (mRNA) of mitochondrial-respiration complexes between the two cell lines (**Figure 3A**). We first verified the protein levels of glycolytic enzymes and respiration complexes *via* WB. Our results confirmed increased expression of hexokinase 2 (HK2), pyruvate kinase 2 (PKM2; **Figure 3B**), and mitochondrial respiration complex II succinate dehydrogenase subunit B (SDHB), and decreased expression of complex I nicotinamide adenine dinucleotide + hydrogen (NADH) ubiquinone oxidoreductase subunit B8 (NDUFB8) in A549/DDP cells, compared with A549 cells. However, we saw no differences in HK1 (**Figure 3B**), complex III ubiquinol-cytochrome c (cyt c) reductase core protein 2 (UQCRC2), complex IV mitochondrially encoded cyt c oxidase I (MT-CO1), or complex V ATP synthase subunit alpha (ATP5A; **Figure 3C**). These findings not only indicated a preference for aerobic glycolysis, but also for enhanced complex II instead of complex I-dependent respiration in cisplatin-resistant A549/DDP cells.

Next, to make sense of the different preferences for mitochondrial metabolic substrates, we assessed the oxygen consumption rate (OCR) between A549 and A549/DDP cells in a substrate dependency assay. BPTES (3 μM), a glutamine inhibitor, was injected into cells, which were subsequently cultured in non-buffered medium supplemented with glucose (10 mM), pyruvate (1 mM), and glutamine (2 mM). Our results showed that OCR decreased gradually, and there was no difference between the two cell lines. After receiving injections of etomoxir (ETO; 4 μM) and UK5099 (2 μM), two inhibitors of glucose, and the long-chain fatty acid (LCFA), A549/DDP cells showed a lower OCR than A549 cells. We then calculated the glutamine dependency rate for OXPHOS and found that this rate was lower in A549/DDP cells than in A549 cells, as well as that these cells depended more on glucose than glutamine for OXPHOS (**Figure 3D**). To assess the effect of glutamine deprivation on OXPHOS, we assessed OCR once again. The two cell lines were almost indistinguishable in terms of basal and maximal respiration in glutamine-containing medium, while A549/DDP cells showed higher basal (oligomycin, 1.5 μM) and maximal (FCCP, 1.5 μM) OCR than A549 cells during glutamine deprivation for 48 h (**Figure 3E**). We further assessed ATP levels, which were mainly generated from mitochondrial respiration, and they were consistent with OCR values (**Figure 3F**). Therefore, our results showed that cisplatin-resistant cells could better survive and sustain energy supply during glutamine deprivation partly because of the dominant effect of glucose, instead of glutamine, on OXPHOS.

WB analysis also confirmed protein levels of complexes I, II, and III. The three major [Fe-S] cluster-binding subunits of these respiration complexes were notably decreased in A549 cells, which may be attributable to decreased ATP generation. Complexes I and III, but not complex II, decreased in A549/DDP cells (**Figure 3G**). Collectively, these findings demonstrate that cisplatin-resistant cells adapted themselves to sustain glucose-dominant complex II-dependent mitochondrial respiration in glutamine-restricted condition.

Succinate dehydrogenase, which functions as complex II in the mitochondrial respiratory chain, is a complex made up of SDHA, SDHB, SDHC, and SDHD subunits. SDHA couples the oxidation of succinate to fumarate with the reduction of covalently bound FAD^+^ to FADH_2_. Three iron-sulfur (Fe-S) clusters in SDHB facilitate the transfer of electrons from FADH_2_ to ubiquinone, which is bound *via* the membrane-embedded SDHC and SDHD subunits (25, 26). To determine the importance of SDHB in A549/DDP cells, we utilized a SDHB targeting siRNA and a non-targeting negative control siRNA (si-Control). Knockdown of SDHB was confirmed by immunoblot analysis in A549/DDP cells in glutamine-containing or glutamine-deprived medium. Using specific siRNA against SDHB, we reached approximately 10% reduction in cell viability compared to the control siRNA in glutamine-containing medium. In addition, the interference of SDHB decreased cell viability by approximately 30% in glutamine-deprived medium (**Figure 3H**). Finally, survival analysis using Kaplan-Meier (KM) estimates indicated that elevated SDHB expression was correlated with a shorter five-year survival rate in 718 lung adenocarcinoma patients (HR = 1.53, *p* < 0.000, **Figure 3I**). Collectively, these findings demonstrate that complex II-dependent OXPHOS drives the adaptive response during glutamine deprivation, and the SDHB subunit can be exploited as an indicator of vulnerability to drug resistance in lung cancer.

### Cisplatin-Resistant Lung Adenocarcinoma Cells Reprogram Iron Metabolism While Becoming Vulnerable to DFO During Glutamine Deprivation

**Figure 4:**
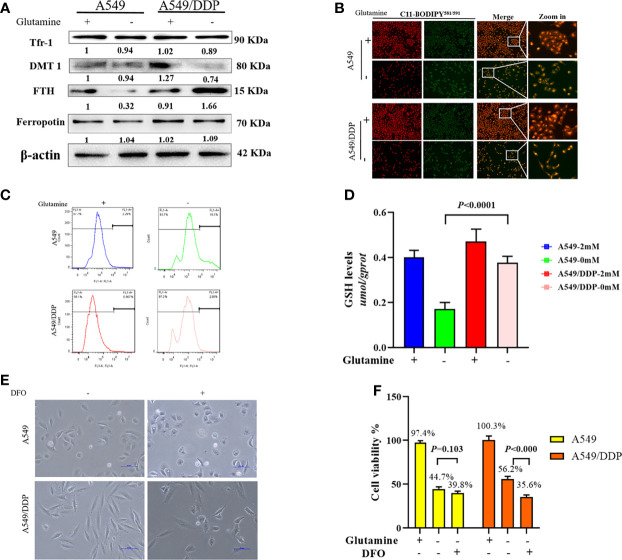
A549/DDP cells reprogrammed iron metabolism and became vulnerable to DFO. **(A)** Blots of iron uptake and storage protein expression in A549 and A549/DDP cells cultured with (+) or without (−) glutamine for 48 h. β-actin served as a loading control. **(B)** Representative images of A549 and A549/DDP cells loaded with C11-BODIPY^581/591^ and cultured with (+) or without (−) glutamine for 48 h. **(C)** ROS levels in A549 and A549/DDP cells cultured with (+) or without (−) glutamine for 48 h. **(D)** GSH concentrations in A549 and A549/DDP cells cultured with (+) or without (−) glutamine for 48 h. **(E)** Morphological observation of DFO treatment’s (100 μM; 48 h) effects on A549 and A549/DDP cells cultured with (+) or without (−) glutamine for 48 h. **(F)** Viability after DFO treatment (100 μM; 48 h) of A549 and A549/DDP cells cultured with (+) or without (−) glutamine for 48 h. All experiments were repeated at least three times, and results were expressed as mean ± SD.

In respiring cells, iron plays crucial roles in the synthesis of [Fe-S] clusters and drives electron transfer pathways in the mitochondrial respiratory complexes (21, 27, 28). To differentiate the role of iron metabolism between the two cell lines, we explored the effect of glutamine deprivation on iron homeostasis. As shown in **Figure 4A**, we found no difference in Tfr-1, FTH, and Fpn protein expression between the two cell lines in glutamine-containing medium. However, the expression of the iron importer DMT1 was higher in A549/DDP than A549 cells, implying an increase in iron uptake to meet elevated respiratory demands in the former. Notably, glutamine deprivation downregulated the expression of FTH protein, but not the expression of other iron metabolism-related proteins in A549 cells; in contrast, glutamine deprivation upregulated FTH and downregulated DMT1 protein expression in A549/DDP cells, except for Tfr-1 and Fpn. Protein-controlled iron homeostasis is essential for maintaining cell growth. Recently, two iron chaperones have been discovered that direct iron within two unique pathways: mitochondrial [Fe-S] cluster assembly and the FTH iron storage system (21, 29). When maintained at a balanced level, iron is essential for cell proliferation, but is toxic when in excess, leading to membrane lipid peroxidation potentiated by reactive iron (Fe^2+^) through Fenton reactions. Therefore, we next performed a lipid peroxidation probe using a sensor. During oxidation, fluorescence shifts from red to green (30). Notably, during glutamine deprivation, the fluorescence intensity of C11-BODIPY^581/591^ was lower in A549/DDP cells than A549 cells (**Figure 4B**). The ROS measurement was also lower (**Figure 4C**). GSH is a major endogenous antioxidant derived from intracellular glutaminolysis (31). We observed that glutamine deprivation decreased GSH levels in both cell lines, but A549/DDP cells showed higher GSH concentrations than A549 cells (**Figure 4D**). Therefore, our data indicated that cisplatin-resistant cells reprogrammed iron metabolism under glutamine deprivation, which might be responsible for respiration and antioxidation reactions.

Based on these findings, we next investigated the effect of DFO treatment on cisplatin-resistant cells. DFO has been shown to promote cell death in cancer cells (32–34). As expected, we found that in A549/DDP cells, cell structures were clearly destroyed, and fewer cells survived after DFO treatment (100 μM; 48 h) during glutamine deprivation for 48 h, but the same was not true of A549 cells (**Figures 4E, F**). Collectively, these findings demonstrate that cisplatin-resistant cells reprogrammed iron metabolism and became vulnerable to DFO treatment during glutamine deprivation.

### DFO Destabilizes Iron Metabolism, Impairs Mitochondrial Respiration, and Induces Oxidative Stress-Mediated Cell Death in Cisplatin-Resistant Lung Adenocarcinoma Cells During Glutamine Deprivation

**Figure 5:**
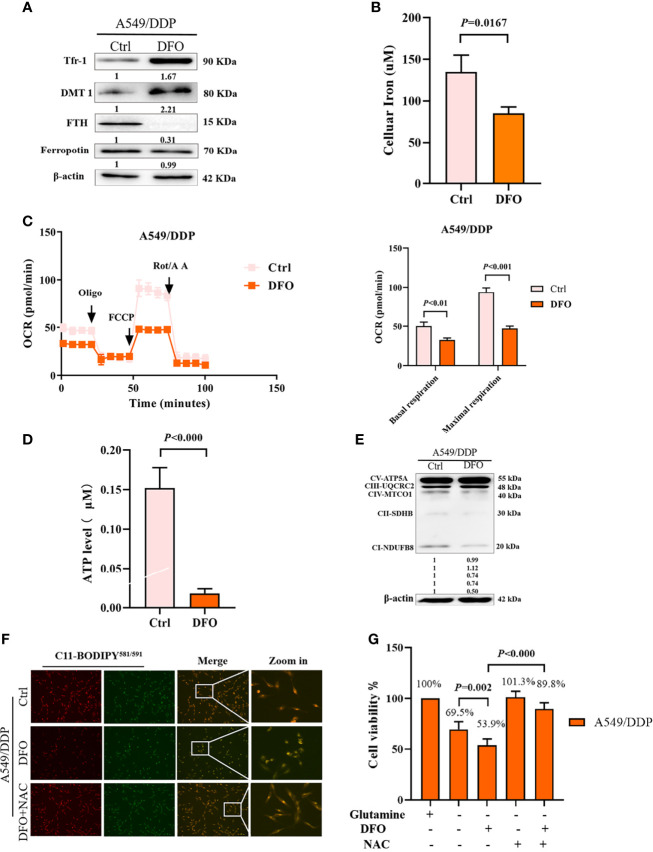
DFO destabilizes iron metabolism, impairs mitochondrial respiration, and induces oxidative-stress–mediated cell death in A549/DDP cells during glutamine deprivation. Effects of DFO (100 μM; 48 h) on: **(A)** iron uptake and storage-related protein expression in A549/DDP cells during glutamine deprivation for 48 h; **(B)** cellular iron concentration in A549/DDP cells during glutamine deprivation for 48 h; **(C)** ETC-dependent oxygen consumption as represented by basal and maximal respiration measured by Seahorse XF assay in A549/DDP cells during glutamine deprivation for 48 h; **(D)** ATP levels of A549/DDP cells cultured in a glutamine-restricted condition for 48 h; and **(E)** on respiration complex protein expression in A549/DDP cells cultured without glutamine for 48 h. **(F)** Effects of DFO (100 μM; 48 h) and NAC (5 mM; 48 h) on lipid peroxidation as represented by C11-BODIPY^581/591^ fluorescence in A549/DDP cells during glutamine deprivation for 48 h. **(G)** Viability of A549/DDP cells after DFO (100 μM; 48 h) and NAC (5 mM; 48 h) treatment during glutamine deprivation for 48 h. All experiments were repeated at least thrice, and the results are expressed as mean ± SD.

Several studies have reported that DFO can significantly affect intracellular and extracellular iron levels, eliminate biologically active iron, impair mitochondrial respiration and biogenesis of [Fe-S] clusters, suppress cell proliferation, and cause tumor cell death (34, 35); however, its effects and mechanisms in cisplatin-resistant LAD cells remain uncertain. Therefore, we investigated the effect of DFO on cellular iron metabolism in A549/DDP cells. In line with these previous studies, our results showed that DFO treatment (100 μM; 48 h) significantly increased TfR-1 and DMT1 protein expression while decreasing FTH protein expression in A549/DDP cells during 48 h of glutamine deprivation (**Figure 5A**). Intracellular iron concentration was determined by the iron colorimetric assay kit. Our results showed decreased intracellular iron concentration after DFO treatment (100 μM; 48 h; **Figure 5B**). Together, these data suggested that DFO destabilized iron metabolism in cisplatin-resistant cells during glutamine deprivation.

Because the function of the electron transfer chain (ETC) highly relies on Fe-S clusters (27, 36), we next assessed the effect of DFO on ETC-dependent oxygen consumption. Our results showed that DFO treatment (100 μM; 48 h) significantly suppressed mitochondrial respiration in A549/DDP cells during glutamine deprivation for 48 h (**Figure 5C**), which was also confirmed by ATP generation (**Figure 5D**). Efficient ETC-linked respiration mainly relies on the assembly of respiratory complexes. Given the profound effect of DFO on mitochondrial respiration, we next analyzed protein levels of respiratory complexes, which were consistent with OCR values: DFO treatment (100 μM; 48 h) lowered both complex I- and complex II-dependent respiration (**Figure 5E**).

The intracellular labile-iron pool directly catalyzes the generation of ROS through Fenton reactions (36). To evaluate oxidative stress (OS) in A549/DDP cells treated with DFO, we tested ROS activity using a C11-BODIPY^581/591^ probe. Notably, DFO treatment (100 μM; 48 h) induced an increase in C11-BODIPY^581/591^ fluorescence intensity; in other words, it induced lipid peroxidation in A549/DDP cells during glutamine deprivation for 48 h. Meanwhile, the ROS scavenger N-acetyl-L-cysteine (NAC; 5 mM; 48 h) rescued DFO-induced lipid peroxidation (**Figure 5F**) and could also fully rescue DFO (100 μM; 48 h)–induced cell death in A549/DDP cells during 48 h of glutamine deprivation (**Figure 5G**). These results suggested that OS was involved in DFO-induced cell death. Taken together, these data demonstrated that DFO destabilized iron metabolism, impaired mitochondrial respiration, and induced OS-mediated cell death in cisplatin-resistant LAD cells during glutamine deprivation.

### DFO Induces Autophagic Cell Death and Apoptosis by Activating ROS-Mediated JNK Signaling

**Figure 6:**
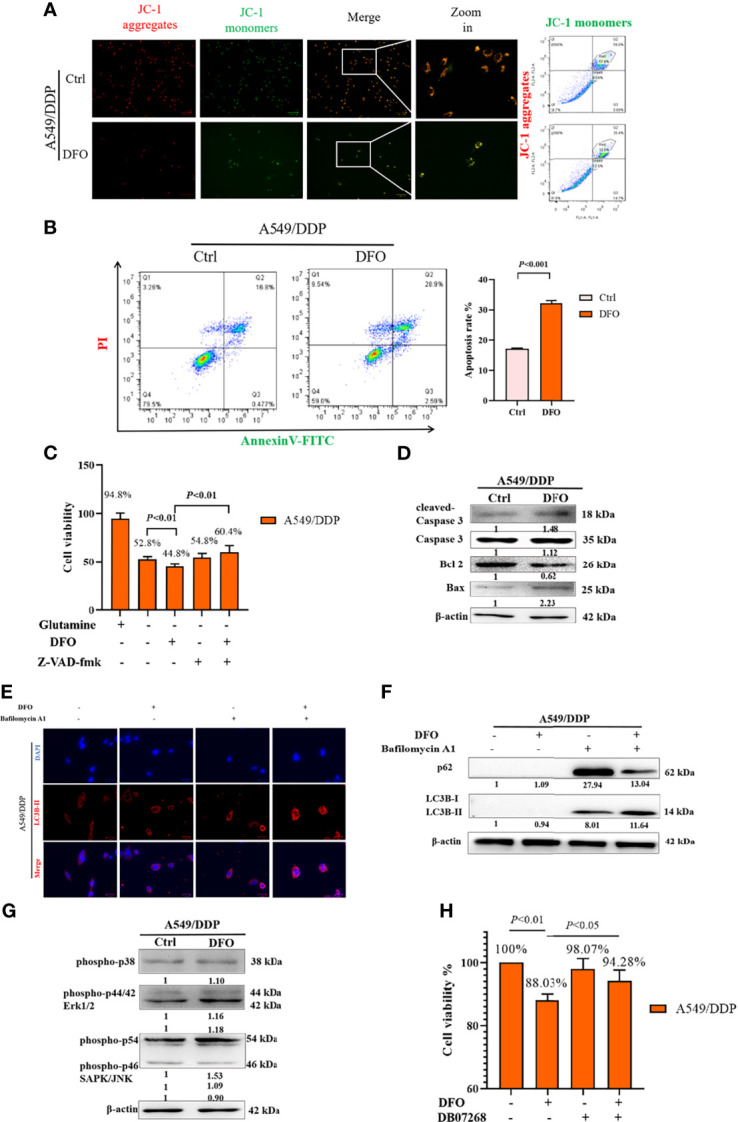
DFO induces autophagic cell death and apoptosis by activating ROS-mediated JNK signaling in A549/DDP cells during glutamine deprivation. Effects of DFO (100 μM; 48 h) on: **(A)** MMP as measured by JC-1 probe in A549/DDP cells during 48 h glutamine deprivation; **(B)** apoptosis in A549/DDP cells during glutamine deprivation for 48 h; **(C)** viability of A549/DDP cells after Z-VAD-fmk (5 µM; 48 h) treatment during glutamine deprivation for 48 h; **(D)** apoptosis-related protein expression in A549/DDP cells during 48 h glutamine deprivation; **(E)** immunostaining after BA1 treatment (10 nM; 48 h) of LC3B A549/DDP cells cultured without glutamine for 48 h (red = LC3B, blue = DAPI); **(F)** autophagic flux after BA1 treatment (10 nM; 48 h) in A549/DDP cells cultured in glutamine-restricted conditions for 48 h, with β-actin serving as a loading control. **(G)** Blots of MAPK signaling in DFO-treated (100 μM; 48 h) A549/DDP cells during 48 h glutamine deprivation; β-actin served as a loading control. **(H)** Viability of A549/DDP cells after DFO (100 μM; 48 h) and JNK-IN-8 (10 nm; 48 h) treatment during glutamine deprivation for 48 h. All experiments were repeated at least thrice, and the results are expressed as mean ± SD.

ROS serve as executioners of programmed cell death (37, 38). To verify the mechanism underlying DFO-induced cell death in A549/DDP cells, we first performed an MMP assay with a JC-1 probe. Our results showed a higher rate of generation of JC-1 monomers and lower rate of generation of aggregates after DFO treatment (100 μM; 48 h) in A549/DDP cells subjected to glutamine deprivation for 48 h (**Figure 6A**), which indicated a loss of MMP. Such loss is regarded as an early event in apoptosis (38). Next, we performed an Annexin V-FITC/PI apoptosis detection assay *via* FCM to assess apoptotic rates; our results showed it to be notably increased in A549/DDP cells after DFO treatment (100 μM; 48 h; **Figure 6B**). We also found that the apoptosis inhibitor Z-VAD-fmk (5 µM; 48 h) could rescue DFO-induced cell death in A549/DDP cells (**Figure 6C**). To further confirm the role of apoptosis in DFO (100 μM; 48 h)–induced cell death, we performed WB to evaluate apoptotic profiles. We found that DFO (100 μM; 48 h) elevated pro-death protein *Bax* expression while reducing anti-death protein *Bcl-2* expression, which triggered activation of Caspase-3 in A549/DDP cells during glutamine deprivation (**Figure 6D**). Collectively, these results indicated that cisplatin-resistant cells underwent apoptosis after DFO treatment. We also observed an increase in autophagic flux: after blockage of the late stage of autophagy by BA1 DFO (100 μM; 48 h) notably increased LC3B puncta (**Figure 6E**) as well as LC3B-II protein expression (**Figure 6F**). Therefore, our results indicated that DFO treatment induced apoptosis and activated autophagy in cisplatin-resistant LAD cells during glutamine deprivation.

DFO reportedly induces apoptosis in leukemic, gastric-cancer, or osteosarcoma cells by activating the MAPK pathway (39–41). MAPKs, including p38, JNK, and ERK1/2, have been implicated in the regulation of apoptosis and autophagy (38). Therefore, we assessed whether MAPK signaling was involved in DFO’s effects on A549/DDP cells. We found that DFO activated the MAPK signaling pathway by phosphorylating JNK, but we observed no changes in p-p38 and p-ERK protein expression in response to DFO treatment (**Figure 6G**). Furthermore, treatment with the JNK inhibitor JNK-IN-8 (10 nm; 48 h) promoted cell viability compared with DFO treatment alone in A549/DDP cells during glutamine deprivation (**Figure 6H**). Therefore, our results indicated that DFO induced apoptosis and autophagic cell death by activating ROS-mediated JNK signaling in cisplatin-resistant LAD cells during glutamine deprivation.

### DFO Inhibits Cisplatin-Resistant Lung Adenocarcinoma Tumor Growth

**Figure 7:**
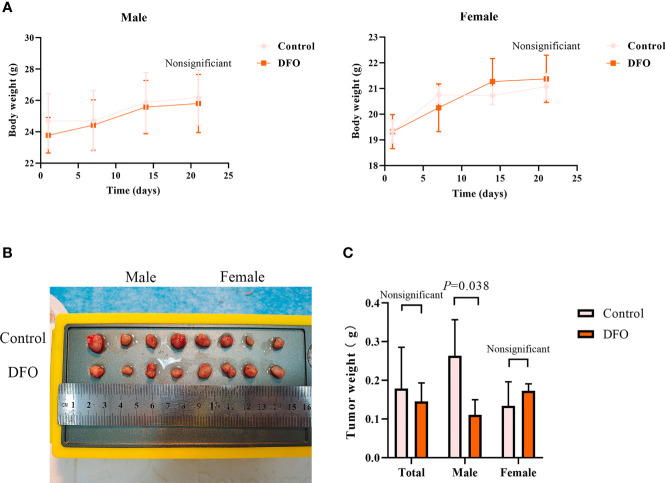
DFO inhibits cisplatin-resistant lung adenocarcinoma tumor growth. **(A)** Body growth curves of mice injected subcutaneously with A549/DDP cells and treated with DFO (8 mg/kg, 2 doses/week for 3 weeks). **(B)** The overall scope of tumors of mice injected subcutaneously with A549/DDP cells and treated with DFO (8 mg/kg, 2 doses/week for 3 weeks). **(C)** Tumor weight of mice injected subcutaneously with A549/DDP cells and treated with DFO (8 mg/kg, 2 doses/week for 3 weeks).

Our *in vitro* studies provide a solid evidence that DFO is effective in inducing cisplatin-resistant LAD cells death. Therefore, we next tested its efficacy *in vivo* using a xenograft mice model. There were no differences in body weight or tumor weight between PBS (Control) and DFO (8 mg/kg, i.p.) treatment groups (**Figures 7A, B**). A growth-slowing effect from DFO was observed in the male, but not female, subgroup, compared to those in the control group (**Figures 7B, C**), indicating the inhibitory effect of DFO on the growth of cisplatin-resistant LAD cells *in vivo*.

## Discussion

Despite numerous studies shedding light on the mechanisms underlying phenotype resistance to cisplatin in NSCLC, the high incidence of cisplatin chemoresistance remains the main limitation of its clinical usefulness (2, 4, 5, 7). Therefore, the development of a chemosensitization strategy is imperative in clinical practice. Glutamine, like glucose, is a major nutrient consumed by cancer cells; pathways involved in glutaminolysis have been exploited for therapeutic purposes (42–44). Pharmacologically targeting glutamine metabolism or withdrawal does not always induce cancer cell death, owing to the cells’ adaptive responses (1, 42, 45). The underlying mechanism targeting glutamine in cisplatin-resistant NSCLC therefore must be fully elucidated.

In the present study, we began by confirming cisplatin resistance. Next, we showed that glutamine deprivation unequally altered cell morphology and viability in two LAD cell lines in a time-dependent manner. A549/DDP cells showed elongated morphology and increased counts compared with those in A549 cells at 48 h, consistent with previous reports (1, 2). Our present findings implied that cisplatin-resistant cells not only attained phenotype resistance to cisplatin but also acquired adaptive mechanisms during glutamine deprivation.

It should be noted that there were numerous drifting and dead cells in the medium after glutamine deprivation for 48 h, especially A549 cells, but fewer A549/DDP cells (data not shown). This prompted us to consider the different roles of glutamine deprivation–induced programmed cell death in two cell lines. First, we measured the apoptotic rate. Our studies uncovered apoptosis resistance characteristics of cisplatin-resistant cells during glutamine deprivation. Autophagy plays a great variety of physiological and pathophysiological roles, including regulation of cell death, proliferation, inflammation, and numerous diseases (46, 47). Generally, autophagy protects cells from stress and blocks the induction of apoptosis. However, in certain cases, autophagy or autophagy-relevant proteins can facilitate activation of apoptosis (48, 49). Meanwhile, studies have generated conflicting findings on whether glutamine starvation induces or inhibits autophagy (15, 48). An alternative explanation for opposite results might be the use of different cell types in studies, such as normal *versus* cancerous epithelial cells. Another reason could be the endpoint of measurement; one study shows that glutamine deprivation induced autophagy in IPec-1 cells with the peak value at 8 h, while subsequently decreasing it (48). In our study, we found that 48 h glutamine deprivation induced autophagy, as autophagic flux increased in both cell lines after 48 h glutamine deprivation and after BA1 treatment. In addition, autophagic flux was higher in cisplatin-resistant cells than that in cisplatin-sensitive cells, which suggests a protective role for autophagy during glutamine deprivation.

Several mechanisms have been proposed to explain how glutamine depletion induces cell death. A reasonable interpretation is association with the fate of glutamine metabolism. As it enters cells *via* transporters, glutamine is converted to glutamate, and then to α-ketoglutarate (α-KG), which enters the Krebs cycle to generate ATP through production of NADH and flavin adenine dinucleotide 2 (FADH2) for OXPHOS (26, 50, 51). Therefore, glutamine might support cell viability by enabling OXPHOS. This hypothesis is consistent with the observation that human pluripotent stem cells (hPSCs) highly rely on glutamine oxidation for ATP generation (52). Cancer cells have been shown to prefer glycolysis to OXPHOS, which is known as the Warburg effect (53, 54). It is also reported that chemo-resistant ovarian-cancer cells can switch between OXPHOS and glycolysis, suggesting an adaptability associated with chemoresistance (55). Our previous study reported that cisplatin-resistant NSCLC cells had a higher glycolysis rate than cisplatin-sensitive cells, characterized by elevated levels of key enzymes (3, 16). In the current study, we validated the differences between key glycolytic enzymes and OXPHOS complexes in cisplatin-sensitive A549 and cisplatin-resistant A549/DDP cells. We confirmed that A549/DDP cells showed higher glycolysis levels than A549 cells, characterized by elevated levels of key enzymes that modulate glycolysis at both mRNA and protein levels. We also observed a difference in OXPHOS between the two cell lines, as indicated by enhanced complex II-dependent and reduced complex I-dependent respiration in cisplatin-resistant cells. To make sense of the preference for OXPHOS in metabolic substrates, we assessed OCR profiles between the two cell lines using a substrate dependency assay. We observed that cisplatin-resistant cells depended more on glucose than glutamine for OXPHOS compared with cisplatin-sensitive cells. Mitochondrial stress tests confirmed sustained OXPHOS in A549/DDP cells compared with A549 cells during glutamine deprivation, and ATP contents were also in accord with OCR values. Therefore, our results supported the idea that cisplatin-resistant cells could better sustain cell survival and energy supply during glutamine deprivation partly because of the predominant role of glucose *versus* glutamine in OXPHOS. In addition, WB analysis also confirmed that protein levels of complexes I, II, and III, three major iron-sulfur [Fe-S] cluster-binding subunits of mitochondrial complexes, were downregulated significantly in A549 cells during glutamine deprivation. This might have contributed to the decrease in OXPHOS activity. Meanwhile, complexes I and III, but not complex II, decreased in A549/DDP cells. To determine the importance of succinate dehydrogenase, we investigated whether cisplatin-resistant A549/DDP cells could be targeted by the siRNA downregulation of SDHB. First, the interference of SDHB reduced cell viability regardless of the use of glutamine-containing or deprived medium for cisplatin-resistant A549/DDP cells; however, this effect was significantly higher when glutamine was depleted. Collectively, these findings demonstrate that complex II-dependent OXPHOS drives the adaptive response and can be exploited as a vulnerability index for drug resistance in lung cancer.

In respiring cells, elemental iron plays crucial roles in the biosynthesis of [Fe-S] cluster co-factors and drives electron transfer pathways in the mitochondrial respiratory complexes. Except for that incorporated into mature red blood cells, the majority of iron is directed toward the iron storage protein FTH or Fe-S clusters (20, 23, 32, 33, 56). The sustained complex II-dependent respiration in A549/DDP cells in glutamine-restricted conditions implied a shift in iron fate; therefore, we next explored the effect of glutamine deprivation on iron homeostasis. DMT1 and TfR-1 are two major transporters for iron entrance into mammalian cells during the transferrin cycle (57). Iron uptake and dependence are enhanced in cancer stemlike cells (CSCs) (23). In the current study, we showed that cisplatin-resistant cells were more dependent on iron: we found higher DMT1 protein expression in A549/DDP than in A549 cells in glutamine-containing medium, despite undifferentiated TfR-1 protein expression. FTH carries a ferroxidase activity that allows storage of ferric hydroxides (Fe^3+^) instead of reactive ferrous iron (Fe^2+^) and can sequester iron and prevent the formation of oxygen free radicals (29). Repression of FTH synthesis is correlated with enhanced sensitivity to OS; conversely, overexpression of FTH has been linked to enhanced cellular protection against oxidant-induced cytotoxicity. Our results showed equivalent FTH protein expression in A549 and A549/DDP cells in glutamine-containing medium, while A549/DDP cells exhibited a flexible adjustment mechanism for iron uptake and storage during glutamine deprivation. Therefore, our data indicated that cisplatin-resistant cells reprogrammed iron metabolism under glutamine deprivation. We hypothesized that this reprogramming might be partly responsible for the decrease in reactive ferrous iron–induced oxidative damage to cisplatin-resistant cells. As expected, we observed that glutamine deprivation induced lipid peroxidation and decreased GSH levels in both cell lines, which was consistent with a report that inhibition of glutamine uptake targeting solute carrier family 1, member A5 (SLC1A5) reduced GSH levels, Krebs cycle activity, and inhibition of OXPHOS in leukemia stem cells (58). To take advantage of this iron metabolism reprogramming ability, we investigated whether DFO could suppress the growth of cisplatin-resistant A549/DDP cells. DFO was originally developed to primarily treat diseases related to iron overload (59). However, in recent years, its therapeutic potential in cancer treatment has emerged. Many studies have shown that DFO plays a pivotal role in the treatment of breast and ovarian cancer (33, 60). Similarly, we found that DFO significantly inhibited cell viability in cisplatin-resistant NSCLC cells during 48 h glutamine deprivation but had only a modest effect on cisplatin-sensitive cells. Thus, cisplatin-resistant cells became vulnerable to DFO treatment during glutamine deprivation.

Next, we confirmed the inhibitory effect of DFO treatment on iron metabolism and mitochondrial respiration *via* WB and OCR measurements, as well as ATP generation. These findings were consistent with previous reports that DFO suppressed tumor growth and metastasis by impairment of iron-sulfur [Fe-S] cluster/heme biogenesis (33). Again, FTH played major roles in preventing the formation of oxygen free radicals. The significant decline in FTH protein expression prompted us to consider whether DFO treatment triggered ROS accumulation-mediated cell death. As expected, DFO induced lipid peroxidation, which was rescued by the ROS scavenger NAC, in A549/DDP cells during 48 h glutamine deprivation. Meanwhile, NAC could also fully rescue cell death induced by DFO. Therefore, our results clearly showed that DFO treatment induces OS, possibly *via* iron metabolism imbalance. ROS is a well-established mediator of programmed cell death (36). Xue et al. reported that DFO induced ROS-related apoptosis in osteosarcoma (39), whereas Kim et al. reported iron chelator-induced apoptosis *via* the endoplasmic-reticulum stress (ERS) pathway in gastric-cancer cells (40). Wang et al. reported that DFO increased dental-pulp stem cell migration and differentiation *via* ROS-induced autophagy (34). In the intrinsic apoptotic pathway, it has been suggested that BAX insertion into the mitochondrial outer membrane induces loss of MMP, which exacerbates cyt c release and Caspase-3 activation (61). In the present study, our results confirmed that DFO treatment induced apoptosis and autophagic cell death, possibly *via* ROS-mediated JNK signaling. This implied a more changeable and complicated role of autophagy in regulating cell survival, which needs further investigation.

To the best of our knowledge, this is the first study that has demonstrated that iron metabolism reprogramming might be responsible for glucose-dominant, instead of glutamine-dominant, complex II-dependent OXPHOS, rendering cisplatin-resistant NSCLC cells susceptible to cell death by iron chelators in glutamine-restricted conditions. In the present work, our *in vitro* study indicated that targeting mitochondria iron metabolism might be a strategy in the treatment of cisplatin-resistant NSCLC, and we identified the effect of iron chelator DFO *in vivo*, which could build a solid foundation for the proposed research to form the basis for developing a drug aimed at modulating mitochondria iron metabolism as a means to treat lung cancer. However, there are certain limitations that need to be considered.

First, DFO showed a mild inhibitory effect *in vivo* compared to the *in vitro* study; these differences point to the fact that its anti-tumor efficiency could be enhanced, and further investigation is essential to optimize the precision of mitochondrial targeting directivity, which, along with its combination with cisplatin in lung cancer therapy, would expand its clinical applications. A recent report (33) showed that mitochondrially targeted deferoxamine (mitoDFO) considerably suppressed tumor growth both in breast and pancreatic cancer cells, as well as in mice models, which implied us the therapeutic strategy that targeting mitochondria in iron metabolism is a potential strategy in the treatment of cisplatin-resistant NSCLC. Further investigation is required to confirm these conclusions in clinical trials involving CDDP and DFO.

## Conclusions

**Figure 8:**
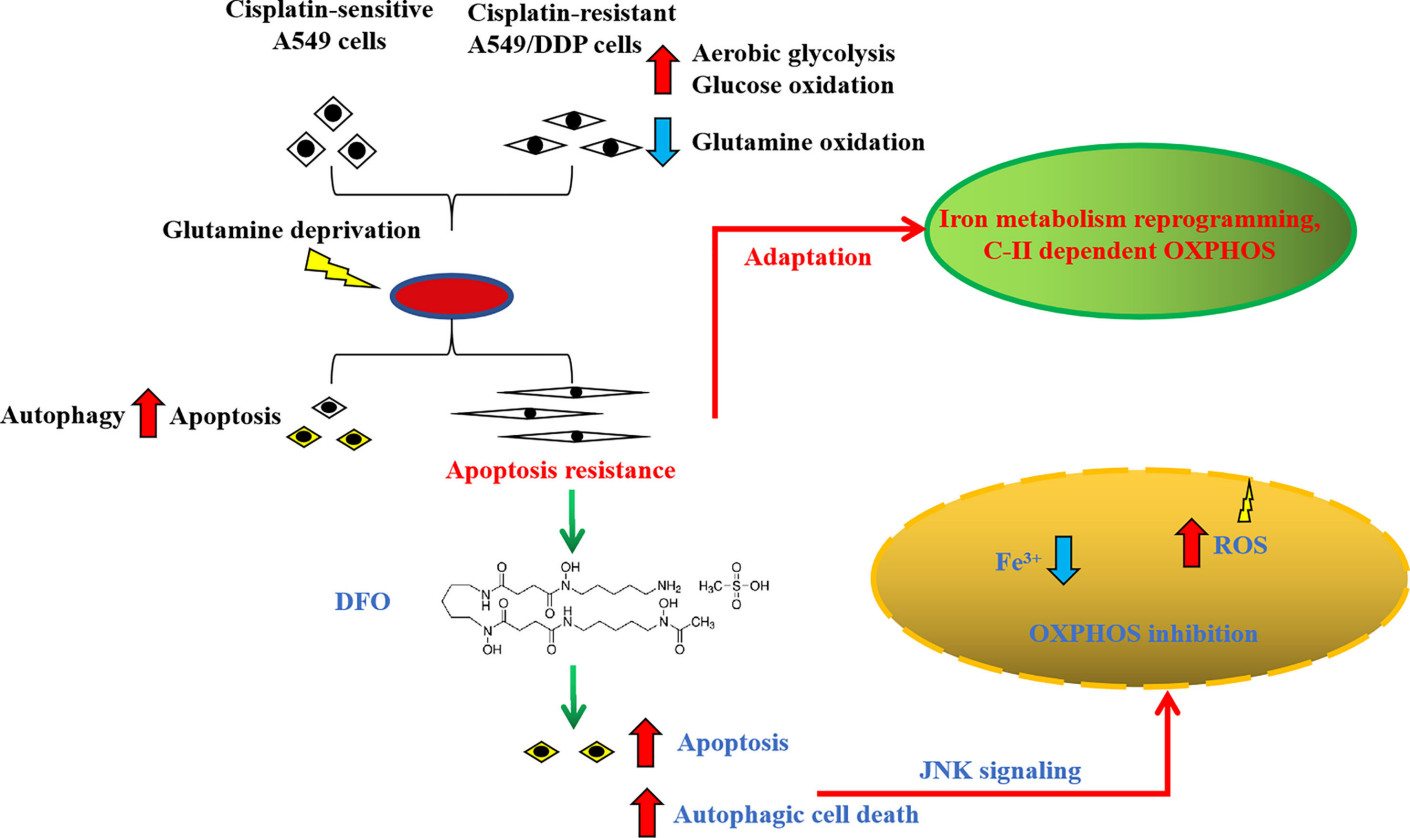
Schematic summarization of the interaction among glutamine deprivation, chemotherapy, and DFO treatment and their effects on JNK signaling-mediated autophagic cell death and apoptosis.

In summary, we found that cisplatin-resistant NSCLC acquired phenotype resistance to cisplatin and adapted themselves to survive in glutamine-deprived conditions, characterized by higher autophagic activity and apoptosis resistance. This adaptation could be explained by glucose-dominant, instead of glutamine-dominant, complex II-dependent OXPHOS. Further investigation revealed that iron metabolism reprogramming might be responsible for mitochondrial [Fe-S] cluster biogenesis during glutamine deprivation, which has become an “Achilles’ heel”, rendering cancer cells susceptible to being killed by DFO treatment induced by OS-mediated autophagic cell death and apoptosis through JNK signaling (**Figure 8**). The *in vivo* study using xenograft mice model also confirmed the growth-slowing effect of DFO. These findings might provide a solid basis of targeting iron metabolism in cisplatin-resistant NSCLC for therapeutic purposes, and it is plausible to consider that DFO could promote the improvement of treatment responses in cisplatin-resistant NSCLC patients.

## Data Availability Statement

The datasets presented in this study can be found in online repositories. The names of the repository/repositories and accession number(s) can be found in the article/supplementary material.

## Ethics Statement

The animal study was reviewed and approved by Animal Core & Welfare Committee of Liaoning University of Traditional Chinese Medicine.

## Author Contributions

L-dZ and CW conceived of and designed this study. W-JL performed the majority of the experiments, analyzed the data, and wrote the manuscript. YS and P-yP conducted the Seahorse assay. J-bW and XX conducted FCM. HZ and Z-yD performed WB and cell viability assays. H-yD conducted animal studies. L-dZ, CW, and W-nC provided funding and experimental platform assistance. All authors have read and approved the final manuscript.

## Funding

This research was supported by the National Natural Science Fund of China (Grants 82174254, 81774184, 81973735, and 81803986), the Education Project of Liaoning Province (Grants L201940), and the Innovation and Entrepreneurship Training Program for college students of Liaoning University of Traditional Chinese Medicine (S202110162011).

## Conflict of Interest

The authors declare that the research was conducted in the absence of any commercial or financial relationships that could be construed as a potential conflict of interest.

## Publisher’s Note

All claims expressed in this article are solely those of the authors and do not necessarily represent those of their affiliated organizations, or those of the publisher, the editors and the reviewers. Any product that may be evaluated in this article, or claim that may be made by its manufacturer, is not guaranteed or endorsed by the publisher.
